# Nanoemulsion Structural Design in Co-Encapsulation of Hybrid Multifunctional Agents: Influence of the Smart PLGA Polymers on the Nanosystem-Enhanced Delivery and Electro-Photodynamic Treatment

**DOI:** 10.3390/pharmaceutics11080405

**Published:** 2019-08-11

**Authors:** Urszula Bazylińska, Julita Kulbacka, Grzegorz Chodaczek

**Affiliations:** 1 Faculty of Chemistry, Wroclaw University of Science and Technology, 50-370 Wroclaw, Poland; 2 Department of Molecular and Cellular Biology, Faculty of Pharmacy with Division of Laboratory Diagnostics, Wroclaw Medical University, 50-556 Wroclaw, Poland; 3 Łukasiewicz Research Network—PORT Polish Center for Technology Development, 54-066 Wroclaw, Poland

**Keywords:** smart nanocarriers, folic acid, verteporfin, cisplatin, SKOV-3 cells, CHO-K1 cells, electroporation, theranostic cargo, double emulsion approach

## Abstract

In the present study, we examined properties of poly(lactide-*co*-glycolide) (PLGA)-based nanocarriers (NCs) with various functional or “smart” properties, i.e., coated with PLGA, polyethylene glycolated PLGA (PEG-PLGA), or folic acid-functionalized PLGA (FA-PLGA). NCs were obtained by double emulsion (water-in-oil-in-water) evaporation process, which is one of the most suitable approaches in nanoemulsion structural design. Nanoemulsion surface engineering allowed us to co-encapsulate a hydrophobic porphyrin photosensitizing dye—verteporfin (VP) in combination with low-dose cisplatin (CisPt)—a hydrophilic cytostatic drug. The composition was tested as a multifunctional and synergistic hybrid agent for bioimaging and anticancer treatment assisted by electroporation on human ovarian cancer SKOV-3 and control hamster ovarian fibroblastoid CHO-K1 cell lines. The diameter of PLGA NCs with different coatings was on average 200 nm, as shown by dynamic light scattering, transmission electron microscopy, and atomic force microscopy. We analyzed the effect of the nanocarrier charge and the polymeric shield variation on the colloidal stability using microelectrophoretic and turbidimetric methods. The cellular internalization and anticancer activity following the electro-photodynamic treatment (EP-PDT) were assessed with confocal microscopy and flow cytometry. Our data show that functionalized PLGA NCs are biocompatible and enable efficient delivery of the hybrid cargo to cancer cells, followed by enhanced killing of cells when supported by EP-PDT.

## 1. Introduction

Effective nanocarriers (NCs) for cancer treatment need both passive and active targeting approaches to achieve highly specific drug delivery to cancer cells while avoiding rapid clearance by the mononuclear phagocyte system and cytotoxicity to normal cells [1]. Recently, the field of biomedical applications, including drug encapsulation, has raised much interest, in part due to the advancement of the biomaterials and “smart” polymers, which enable preparation of containers with novel functional properties (e.g., size, charge, interfacial functionalization) by means of nanoemulsion structural design. Nanoemulsion systems (so-called submicron emulsions, parenteral emulsions, or miniemulsions) are referred to in the literature as transparent or translucent (often bluish) isotropic dispersions of water and oil, with nano-domains coexisting in high kinetic equilibrium due to the occurrence of surfactant molecules at the oil/water interface [2,3]. Owing to their small size (usually in the range 20–200 nm), high kinetic stability, and much lower surfactant concentration (typically 3–10%) required for their formation in comparison to microemulsions (generally about 20% and higher, both oil-in-water (o/w) and water-in-oil (w/o), nanoemulsions were found to be very attractive for droplet engineering to obtain efficient nanocarriers with functional properties. The usefulness of these formulations as raw structures for container fabrication based on their surface templating approaches, by means of biocompatible polymers and polyelectrolytes, including very effective solvent evaporation and layer-by-layer techniques, have been proved by many scientific investigations [4,5,6,7]. Consequently, the structuration of the nanoemulsion droplets is typically carried out with three general approaches: layering, embedding, and clustering [8]. In the embedding process, the double emulsion method permits encapsulation of both hydrophilic and hydrophobic agents in the double compartment structure [7]. Such a hybrid cargo can be loaded simultaneously or separately in the NC liquid core and protected from the environmental conditions [9]. Additionally, the hybrid molecules may also migrate from the outer to inner phase and form a reservoir that enhances spectroscopic or functional properties of photodynamic therapy (PDT) and leads to synergistic anticancer activity. The biological effectiveness of such multifunctional nanosystems in the combined anticancer therapy may be also intensified by increasing their cellular uptake by the electroporation (EP) approach.

The electroporation method is widely used not only in chemotherapy for drug delivery but also in molecular biology and microbiology for gene transfection. The latest data show combination methods are used after electrochemotherapy, where plasmid electrotransfer is applied for stimulation of the immune response for better recovery [10]. It is also crucial that the electroporation technique significantly shortens the time of exposition to the “therapeutic substance”. This approach is definitely safes then chemical methods because it does not introduce any additional components to the reaction environment. The EP approach was shown to efficiently increase both the uptake and the photodynamic activity of some second-generation photosensitive agents from the cyanine (e.g., AlPcS4) and chlorin (e.g., Ce6) families for PDT application [11]. The beneficial impact of EP was also demonstrated in the case of porphyrin-origin dyes (e.g., CoTPPS and MnTMPyPCl_5_), which were used against drug-resistant breast and colon cancer cells [12]. These and other studies performed with phthalocyanines (AlPc and Pc green) [13] showed the promising effect of EP in overcoming drug resistance. Moreover, electroporation can be used for supported delivery or fusion of nanosystems containing multifunctional cargo, which is not possible with chemical methods. Thus, the EP method was effective not only for drugs and photosensitizers in free form but also for encapsulated ones [14]. Our previous study shows that EP enhances delivery and photodynamic activity of encapsulated cyanine IR-780 combined with flavonoids [15]. Thus, the available data clearly indicate a new trend in the application of multifunctional methods in more specific drug delivery to cancerous cells.

Over the last years, different chemical compounds have been utilized in order to improve the stability of double emulsion. This has included various mixtures of surfactants, two types of emulsifiers, and co-surfactants or copolymers to support the final formulation [16,17]. Nevertheless, as has been recently shown, the formation of the nanoemulsion interface by the solvent evaporation approach using functionalized biopolymers, i.e., polyethylene glycol (PEG)-ylated polyesters, such as poly(d,l-lactic acid) (PDLLA), poly(glycolic acid) (PGA), polycaprolactone (PCL), and poly(lactic-*co*-glycolic acid) (PLGA), enhances the long-term colloidal stability of encapsulated bioactive compounds and extends the functional performance of nanoscopic emulsion formulations, rendering them non-toxic and stable in the bloodstream [7,18]. Furthermore, application of PLGA as one of the most promising Food and Drug Administration (FDA)-approved polymers for human use, for example functionalized by folic acid (FA) molecules with selective affinity to the folate receptor (FR), could be beneficial in treatment of human carcinomas with overexpression of FR, including ovarian, breast, and lung cancer cells [19,20].

Thus, in the present study, we prepared NCs coated with PLGA, PLGA-PEG, or PLGA-FA by means of the double emulsion (water-in-oil-in-water (w/o/w)) evaporation process, which enabled co-encapsulation of a hydrophobic, porphyrin-origin photosensitizing agent—verteporfin (VP) in combination with low-dose cisplatin (CisPt)—a hydrophilic chemotherapeutic drug. Obtained NCs were characterized using a variety of analytical methods and functional assays. Finally, their utility in bioimaging as well as in anticancer applications upon human ovarian cancer SKOV-3 cells and control hamster ovarian fibroblastoid CHO-K1 cells was also analyzed when combined with EP and PDT.

## 2. Materials and Methods

### 2.1. Chemicals and Reagents

Poly(lactide-*co*-glycolide), ester endcap L:G 50:50 (PLGA, Mw ~ 25,000–35,000), poly(ethylene glycol) methyl ether-block-poly(lactide-*co*-glycolide) (PEG-PLGA, Mw ~ 5000:20,000), poly(lactide-*co*-glycolide)-folate, L:G 50:50 (FA-PLGA, Mw ~ 25,000–35,000) employed as biocompatible, “stealth” and functionalized “smart” polymers, respectively, for NC stabilization were obtained from PolySciTech^®^ (West Lafayette, IN, USA). Cremophor A25 and didodecyldimethylammonium bromide, di-C_12_DMAB, applied as a hydrophilic non-ionic and a hydrophobic double chain cationic surfactant, were obtained from BASF Care Creations (Monheim am Rhein, Germany) and Sigma Aldrich (Poznan, Poland), respectively. Verteporfin (VP) and cisplatin (Cis-Pt), utilized as hybrid cargo, were from Sigma-Aldrich (Poznan, Poland). Supplementary chemical compounds were of commercial grade and were used as received. Doubly distilled water was purified using a Milli-Q purification system (Millipore, Bedford, MA, USA).

### 2.2. Co-Encapsulation of Hybrid Agents in Polymeric Nanocarriers by Double Emulsion (w/o/w) Solvent Evaporation Method

Polymeric NCs stabilized by PLGA, PEG-PLGA, FA-PLGA, and non-ionic and cationic surfactants for co-encapsulation of a therapeutic (CisPt), as well as a diagnostic and therapeutic agent—VP (both in the initial concentration of 130 µM)—were obtained using double emulsion (w/o/w) evaporation method [7,21]. Generally, we emulsified an aqueous internal phase (with CisPt) in dichloromethane (with VP, PLGA at a concentration of 5 mg/mL and di-C_12_DMAB) at 1:4 ratio using a homogenizer set to 25,000 rpm for 5 min. Next, the primary water-in-oil (w/o) nanoscopic emulsion was poured into 1% Cremophor A25 aqueous solution (a hydrophilic surfactant), stirred in a homogenizer for 10 min (25,000 rpm), and immersed in an ice-water bath to obtain the w/o/w emulsion. Then, we evaporated the organic solvent under reduced pressure in a rotary evaporator (Hei-VAP Value Digital, Heidolph Instruments, Schwabach, Germany) with a rotation speed of 150 rpm for 30 min at 25 °C and polymeric NCs with a PLGA, PEG-PLGA, or FA-PLGA shell. The hybrid cargo were collected the following day.

### 2.3. Nanocarrier Size, Polydispersity, and Particle Charge

The main physicochemical parameters of NCs, such as hydrodynamic diameter (D_H_), polydispersity index (PDI), and particle charge (ζ-potential), were analyzed by means of dynamic light scattering (DLS) and microelectrophoretic methods using Zetasizer Nano Series (Malvern Instruments, Worcestershire, UK) equipped with a He–Ne laser (632.8 nm). DLS measurements were conducted at 25 °C and the detection angle was 173°, as previously described [7,21,22]. Each value was an average of three runs, with at least 10–20 measurements. We applied the DTS (Nano) program for data evaluation.

### 2.4. Shape and Morphology

The morphology of the obtained NCs was studied by atomic force microscopy (AFM) and transmission electron microscopy (TEM) according to our previous protocols [7,21]. The AFM observations were conducted using a NanoScope Dimension V instrument with an RT ESP tube scanner (Veeco Instruments, Plainview, NY, USA) Samples were analyzed at 0.5 Hz scanning speed using a low-resonance-frequency pyramidal silicon cantilever resonating at 250–331 kHz at a constant force of 20–80 N/m. The resonance amplitude was adjusted manually to the lowest possible amplitude enabling stable imaging within the contamination layer on the surface. We prepared the samples by adsorption of an NC droplet on mica that was freshly cleaved. After 18 h, the excess substrate was removed by rinsing the mica plates in double distilled water for 1 min and drying for 2 h at room temperature. The TEM imaging of NCs was performed with an Field Electron and Ion Company (FEI) Tecnai G2 20 X-TWIN electron microscope (FEI, Brno, Czech Republic) by placing a few drops of diluted NCs on a Cu-Ni grid and leaving the specimens to dry for 20 h at room temperature.

### 2.5. Encapsulation Efficiency

The Ultraviolet - Visible (UV-VIS) absorbance of NCs with encapsulated VP and Cis-Pt was measured with a Metertech SP8001 spectrophotometer with a 1-cm length path thermostated quartz cell in order to evaluate the encapsulation efficiency (*EE*). The hybrid cargo concentration was calculated using calibration curves according to our previous protocol [21,22]. We determined *EE* as follows:

$$EE=\frac{W_{added}-W_{free}}{W_{added}}\times 100\;\%$$

where *W_added_* is the amount of VP or CisPt added during the encapsulation procedure, and *W_free_* is the amount of free cargo in the supernatant quantified by UV-VIS spectroscopy after separation of NCs by centrifugation process (14,000 rpm for 30 min).

### 2.6. Colloidal Stability

The backscattering (BS) of pulsed near-infrared, IR light (l = 880 nm) was utilized to measure the long-term colloidal stability of NCs (Turbi-ScanLabExpert, Formulaction SA, Toulouse, France) [7]. In general, two synchronous optical sensors (transmission and backscattering detectors) recorded light transmitted through the sample (0° from the incident radiation) and light back-scattered by the sample (1358 from the incident radiation). The scanning of the sample was performed in a cylindrical glass cell at 25 °C by moving along the entire height of the cell. The BS profiles as a function of the sample height were then collected and analyzed using the instrument’s software (Turbisoft version 2.0.0.33, Formulaction SA, Toulouse, France). We measured BS for freshly prepared NCs and after 30 days of the sample storage at 25 °C.

### 2.7. Cell Lines

The biological studies were performed on a human ovarian carcinoma cell line resistant to diphtheria toxin, cisplatin, and adriamycin (SKOV-3), and a hamster ovarian fibroblastoid cell line (CHO-K1) used as a model for transport studies in a pulsed electric field due to very low expression of endogenous ionic channels [23]. The SKOV-3 and CHO-K1 cells were purchased from ATCC^®^ (American Type Culture Collection, distr. LGC Standards, Lomianki, Poland), cultured, and prepared according to the conditions described previously by our group [15].

### 2.8. Uptake of Encapsulated Hybrid Cargo—Flow Cytometry Analysis

The ability to internalize free and encapsulated VP/CisPt by CHO-K1 and SKOV-3 cells was analyzed by flow cytometry using fluorescence-activated cell sorter (FACS, Cube-6, SYSMEX EUROPE GmbH, Warsaw, Poland). Cells were harvested on 12-well plates and after obtaining 80% of confluence, appropriate nanosystems were added as follows. Free VP or NCs containing VP/CisPt were added with a final VP concentration equal to 2.0 × 10^−6^ M. Then, the cells were incubated for 24 h at 37 °C in a humidified atmosphere containing 5% CO_2_. In the next step, cells were detached with Trypsin-EDTA (Sigma-Aldrich Merck-Group, Poznan, Poland), washed in PBS, and resuspended in 0.5 mL of PBS. Flow cytometry analysis was performed using a Cube 6 flow cytometer (Sysmex, Warsaw, Poland). The fluorescence of VP was measured with a FL-4-H detector. Data were collected and analyzed by CyView software (Sysmex, Warsaw, Poland).

### 2.9. Electroporation Protocol

Electropermeabilization of cell membranes alone and with free or co-encapsulated VP and Cis-Pt was performed using Gene Pulser Xcell™ Electroporation System (BioRad Laboratories, Warsaw, Poland). When cells reached 80% of confluency they were trypsinized and centrifuged (5 min, 1000 rpm). Then, cells were counted and resuspended in 200 μL of electroporation buffer (EP buffer of low electrical conductivity of 0.14 S/m) at cell concentration of 3 × 10^6^/mL [15]. Cells were maintained in suspension and pulsed in a cuvette (VWR) with two aluminum plate electrodes (4 mm gap). The following parameters of electroporation were applied: electrical field intensity *E*(appl) = 500 V/cm, 5 rectangular unipolar pulses of 1 ms duration. The EP conditions were established according to our previous study [14]. The EP experiments were performed using Gene Pulser Xcell™ Electroporation System 165-2660 (BioRad). After the pulse delivery, cells were incubated for 10 min at 37 °C, then gently centrifuged, resuspended in the cell culture medium (DMEM for SKOV-3 cells or HAM’s F10 for CHO-K1 cells, Sigma-Aldrich Merck-Group, Poznan, Poland), and further analyzed by confocal microscopy and subjected to photocytotoxicity studies.

### 2.10. Intracellular Internalization Studies by Confocal Microscopy

The internalization of co-encapsulated VP by cancer and normal cells was studied with confocal microscopy. Briefly, SKOV-3 and CHO-K1 cells were seeded onto glass cover slips in Petri dishes (Sarstedt - distr. Equimed, Wroclaw, Poland) at a density of 1 × 10^4^ cells per cover slip in a CO_2_ incubator for 24 h. Next, the cells were treated with NCs at a concentration corresponding to 2.0 × 10^−6^ M of VP and incubated at 37 °C for 24 h. For the EP-supported uptake, cells were first processed as described in the Section 2.9 and then seeded onto cover slips, thus the time of exposition to nanosystems was only 10 min. After 24 h, all samples were fixed in 4% formaldehyde (Polysciences Inc., Hirschberg an der Bergstrasse, Germany), washed, and placed onto basic glass slides (SuperFrost, Menzel, Braunschweig, Germany) upon mounting in an anti-fade medium (Roth – distr. Linegal Chemicals Sp. z o.o, Warsaw, Poland) with with 4′,6-diamidino-2-phenylindole (DAPI) for nuclei staining. Microscopy was performed on a spinning disk confocal microscope (Cell Observer SD, Zeiss, Oberchochen, Germany). DAPI was visualized with a 405 nm laser and 450/50 emission filter, while VP fluorescence was excited with a 488 laser and collected with a 629/62 nm emission filter.

### 2.11. Photodynamic Activity Protocol

Photocytotoxicity of NCs in SKOV-3 and CHO-K1 cells was measured after standard photodynamic procedure and PDT combined with the EP protocol described above by cellular mitochondrial activity determined by the (3-(4,5-dimethylthiazol-2-yl)-2,5-diphenyltetrazolium bromide (MTT) colorimetric assay according to the manufacturer’s procedure and our previous experiments [12,13,14,15]. The cells subject to PDT were irradiated after 24 h for 10 min with light in the range of 630–680 nm; the final energy delivered to the cell monolayer was 10 J/cm^2^. The MTT assay was performed after 24 h post irradiation for the NCs loaded with the theranostic cargo at a concentration equivalent to 1.0–5.0 × 10^−6^ M of encapsulated VP. The measurements were performed on the GloMax^®^ Discover multimode microplate reader (Promega, Madison, WI, USA). The cell viability in each group was expressed as a percentage of the value obtained for control (untreated) cells (average of three experiments).

### 2.12. Statistical Analysis

The results are presented as means ± standard deviation (SD) values for minimum *n* = 3 repeats. The results were analyzed by two-way ANOVA for multiple comparisons and *α* = 0.05 GraphPad Prism 7.05. The values where *p* ≤ 0.05 (marked with *) were considered as statistically significant.

## 3. Results and Discussion

### 3.1. Characteristic of “Smart” PLGA Nanocarriers Obtained by Nanoemulsion Structural Design

The combination of biomaterials, “smart” polymers, and drug delivery systems for both therapeutic and diagnostic (theranostic) approaches enables the development of intelligent devices and brings enormous possibilities for biomedical applications [24]. Biodegradable polyesters, such as poly(glycolic acid) (PGA), poly(lactic acid) (PLA), and their copolymers are the favorite synthetic polymers for biomedical and pharmaceutical applications, since they were proved to be useful in the stabilization of different drug delivery systems and have excellent biocompatibility and bioresorbability [25].

**Scheme 1:**
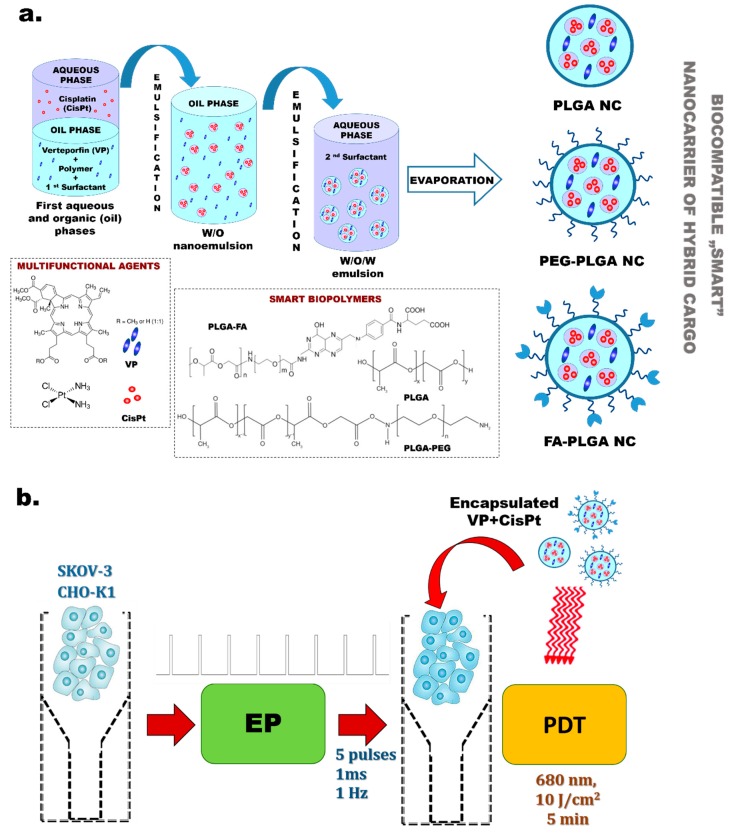
The general strategy of conducted studies: (**a**) double emulsion (w/o/w) design for co-encapsulation of Verteporfin (VP) and Cisplatin (CisPt) in various Poly(Lactide-*co*-Glycolide) (PLGA) nanocarriers (NCs) and (**b**) electroporation (EP)-supported photodynamic therapy (PDT) using obtained NCs against human ovarian cancer (SKOV-3) and hamster ovarian control (CHO-K1) cells.

With this in mind, we have tested highly biocompatible polymers made of PLGA (the FDA-approved copolymer of PGA and PLA, also functionalized with different moieties), namely PEG and FA. The “smart” co-polymers were used to design NCs co-loaded with VP, playing a dual role of a diagnostic and a therapeutic agent, and CisPt, a supporting chemotherapeutic drug. Engineered NCs with encapsulated hybrid cargo had an improved colloidal stability and therapeutic activity, as well as extended functional performance with unique attributes e.g., non-toxicity, stability in blood circulation, and cancer-targeting ability. Thus, according to the first phase of our general strategy presented in Scheme 1a, PLGA, PEG-PLGA, and FA-PLGA polymers were used for stabilization and structuration of nanoemulsion droplets involving the three-step w/o/w double emulsion evaporation approach, leading to co-encapsulation of the hybrid cargo, i.e., VP and CisPt in the NC’s double compartment.

The second step (Scheme 1b) involved EP-supported PDT upon improved internalization of NCs by human ovarian cancer (SKOV-3) and normal ovary fibroblastoid (CHO-K1) cells. The SKOV-3 cells were selected as difficult-to-treat cells, which are extremely resistant to the wide spectrum of cytostatic drugs, but in particular to cisplatin. Consequently, in our study an attempt was made to design special nanosystems to overcome drug resistance phenomena in human ovarian cancer and also very probable secondary resistance to CisPt, and for rapid elimination of the drug from circulation [26]. The PLGA nanocarriers proposed here can significantly diminish this process, causing good bioavailability and the “willingness” of the cell to accept the natural carrier. Furthermore, as has been proved recently, different drug delivery systems with a negatively charged surface are generally less toxic compared to the positively charged ones, but their cellular uptake may be hindered due to the same negative charge present on the surface of target cells. Thus, the electropermeabilization may enhance the transport of theranostic cargo to target (malignant) cells in spite of their negative surface charge [15,27].

**Figure 1:**
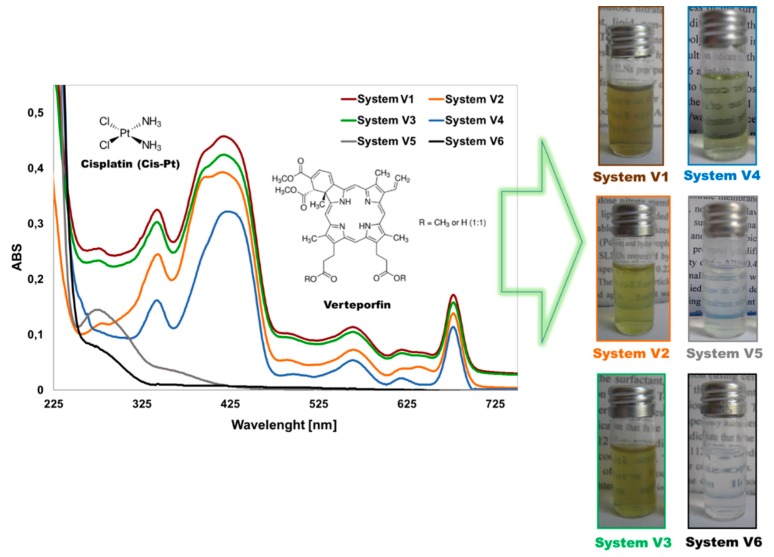
Ultraviolet - Visible (UV-VIS) spectra and images of the VP and CisPt co-loaded nanocarriers with various PLGA shells (samples V1–V3) compared to the control samples loaded only by the cytostatic drug (V5), only by the photosensitizer (V4), and the empty NCs (V6). See descriptions for the systems (V1–V6) in Table 1.

Accordingly, three types of PLGA-NCs loaded with theranostic cargo and three control NCs (systems V1-V6, Table 1) were successfully synthesized. The main physicochemical characteristics of the designed NCs are summarized in Table 1. We measured size (hydrodynamic diameter, D*_H_*), polydispersity index (PdI), zeta potential (ζ), and encapsulation efficiency of VP (EE_VP_) and CisPt (EE_CisPt_). NCs with different PLGA shells displayed an average size between 187 and 200 nm, PDI of approximately 0.1–0.2, and ζ from −4 mV to −17 mV, proving efficient assembly of NCs with PEG-ylated and FA-functionalized shells [7,28]. Generally, loaded cargo did not significantly change the NC charge (ζ), as observed for control NCs with a FA-PLGA shell, loaded with VP, CisPt, or empty ones. Furthermore, the loaded NCs showed only a slightly larger size and less unimodal size distribution, which was probably caused by the incorporated cargo molecules. The EE was about 95% for VP and 90% for CisPt. The differences in the encapsulation of the hybrid cargo by PLGA, PEG-PLGA, and FA-PLGA shells are presented in Figure 1 as UV-VIS spectra of the co-encapsulated VP and CisPt compared to the control samples with only the cytostatic drug and with only the photosensitizer, as well as empty NCs. In all nanosystems a characteristic peak at 280 nm for CisPt as well as peaks at 340 nm, 415 nm, and 680 nm for VP can be observed, providing evidence of effective encapsulation of both ingredients.

**Figure 2:**
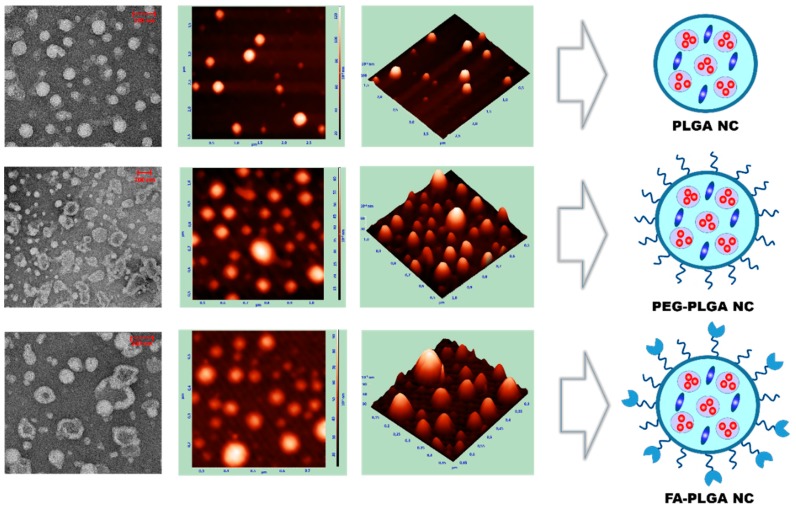
Imaging of VP and CisPt co-loaded PLGA (V1), PEG-PLGA (V2), and FA-PLGA (V3) NCs by means of transmission electron microscopy (TEM) and atomic force microscopy (AFM). See descriptions for the systems (V1–V3) in Table 1.

Meanwhile, the correct imaging of NCs is a key parameter in the design of any drug delivery system dedicated to pharmaceutical applications. The obtained PLGA-stabilized NCs were characterized by transmission electron microscopy (TEM) and atomic force microscopy (AFM)—quick, efficient, and relatively non-invasive techniques that can provide evidence on shape and morphology and size distribution of these polymeric nanosystems. The TEM and AFM images of the loaded PLGA-NCs are shown in Figure 2. The TEM imaging demonstrated spherical particles with roughly uniform sizes related to AFM. Furthermore, we observed some differences in morphology as visualized by TEM for nanocarriers prepared with different PLGA shells. In the case of NCs covered by PLGA, the spherical nanoobjects with relatively smoother surface morphology were discovered by both TEM and AFM imaging, while the NCs stabilized by PEG-PLGA and FA-PLGA (Figure 2) had a typical core shell morphology, where the darkest part relates to the denser polymeric/PEG-ylated corona, demonstrating that these shells were successfully formed. The AFM tapping mode scanning presented as 2D and 3D images identified a semi-spherical shape of the NCs, being slightly less regular in the case of NCs stabilized only by PLGA (Figure 2). However, we did not see increased aggregation as it was found by TEM. The NC’s size range was smaller than the distribution obtained by the DLS measurement presented in Table 1, as both TEM and AFM were carried out in dry conditions, and the obtained NCs have a tendency to shrink, resulting in losing their primary shape and size [29].

### 3.2. Evaluation of Colloidal Stability

The colloidal stability of NCs is one of the most critical factors for any potential biological application, since these nanostructures, when not stabilized electrostatically, are usually metastable due to short-range van der Waals attraction [30]. Consequently, to avoid NC aggregation due to their low colloidal stability, steric or electrostatic repulsion may be applied for stabilization. The literature data indicate the encapsulation of inorganic and organic molecules by surfactants and polymers as the best strategy for the enhancement of any nanostructure colloidal stability and functionality, leading to hybrid core/(polymer-)shell NCs [31]. However, the NC aggregation process is still hard to control, especially in biological environments, which is of essential significance for in vivo applications [6,30].

**Figure 3:**
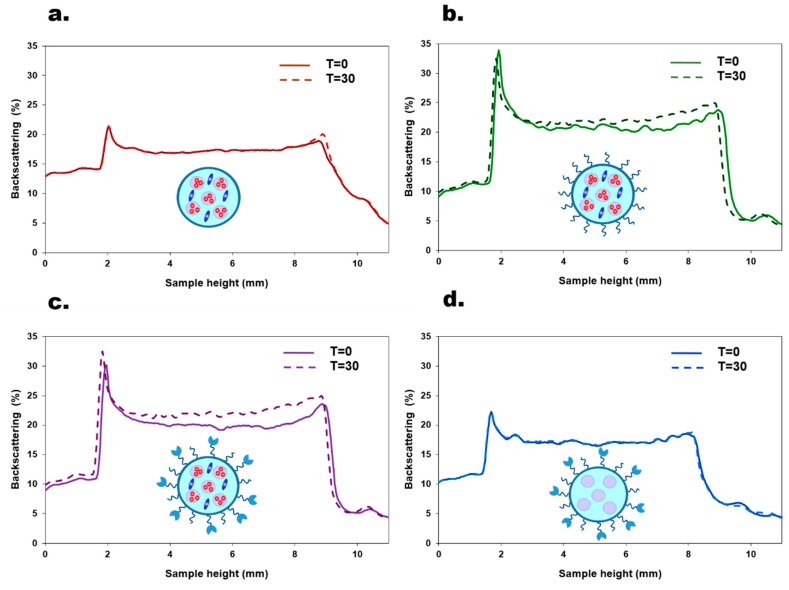
Backscattering profiles of the various PLGA NCs co-loaded with VP and CisPt (**a**–**c**) (V1–V3) and an empty (**d**) (V6) FA-PLGA nanosystem as a function of sample height (mm) analyzed over 30 days (*T* = 30) of the NCs’ storage. Solid lines represent measurement at 0 day (*T* = 0, freshly prepared NCs), while dashed lines indicate data obtained after 30 days of storage. See descriptions of the systems in Table 1.

The detailed estimation of the colloidal stability of different PLGA co-loaded and empty NCs was conducted using turbidimetric method by means of time-dependent BS profiles. The BS levels expressed in % are indicated on the ordinate axis. The investigated formulation level in the measurement vial was expressed in mm and marked on the abscissa axis. By examining the BS profiles (Figure 3), we were able to determine the dynamics of any decomposition processes occurring within the sample. This was achieved through analysis of the distances between the curves in BS profiles of NCs at 0 days (freshly prepared) and after 30 days of their storage at room temperature (Figure 3).

Typically, rapid destabilization phenomena can be recognized by a large distance between the curves, while an overlap of the individual curves indicates the high stability of the analyzed sample and a slow rate of the destabilization process [32]. Based on the graphs shown in Figure 3, we conclude that the studied nanosystems have good colloidal stability, since no macroscopic changes in analyzed samples (aggregation, sedimentation, or creaming processes) were observed at the last day of the performed turbidimetric test.

### 3.3. Cellular Internalization—Flow Cytometry and Confocal Microscopy Evaluation

**Figure 4:**
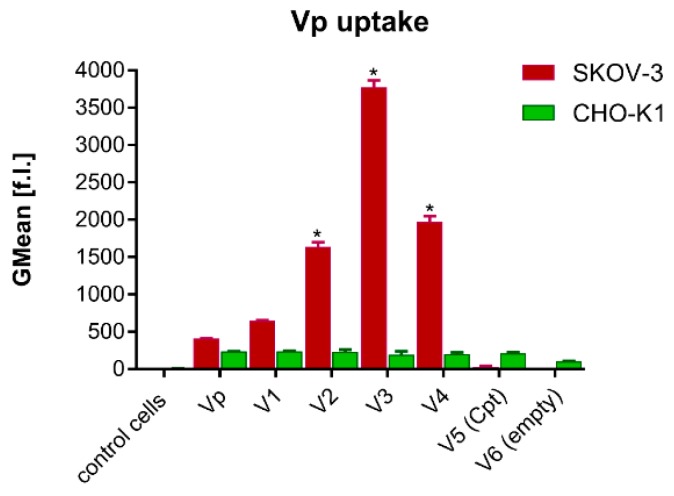
Influence of the nanocarrier surface on the encapsulated cargo uptake by SKOV-3 and CHO-K1 cells evaluated by flow cytometry analysis. The uptake of NCs was expressed as mean fluorescence intensity of VP in the gated cell population after 24 h incubation of cells with the loaded PLGA NCs (V1–V5), control cells (control), empty nanocarriers (V6). and free VP (Vp). See descriptions of the systems in Table 1.

The evaluation of nanosystem uptake by cancer and normal cells is shown in Figure 4. The uptake efficiency of NCs was estimated by flow cytometry after 24 h of exposition. SKOV-3 cells revealed a higher uptake efficiency of VP-loaded NCs than CHO-K1 cells. The strongest fluorescent signal was observed for V3 (VP + CisPt) and V4 (VP) nanosystems, which were functionalized by FA moieties, proving the effective SKOV-3 targeting ability of the “smart” FA-PLGA-coated nanodevices [33]. The improved uptake in the case of nanosystem V3 can be explained by the content of isplaitin, which can slightly sensitize exposed cells and provoke better internalization of VP.

**Figure 5:**
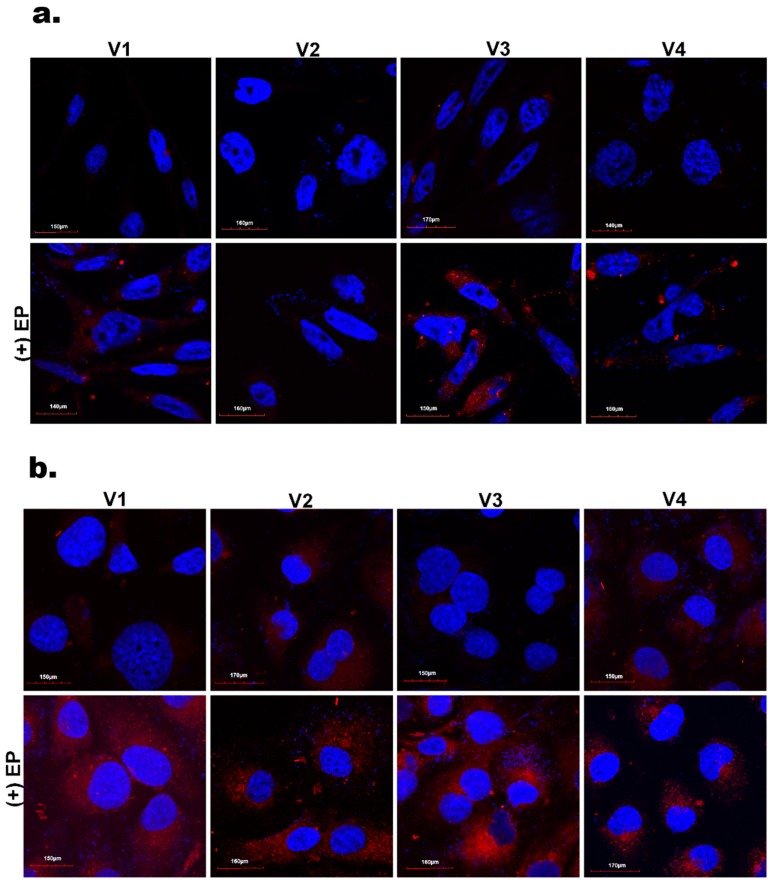
Intracellular distribution of nanosystems after 24 h incubation with NCs (upper panel) or 10 min exposition to NCs after EP (lower panel) in (**a**) CHO-K1 cells and (**b**) SKOV-3 cells.

The confocal microscopy was conducted on cells treated with NCs and electroporated. The exposition time to treatment was much shorter and did not exceed 10 min. The acquired microphotographs are presented in Figure 5. Upon EP, the uptake of encapsulated drugs increased, in particular for FA-PLGA-coated NCs (V3 and V4) in both cell lines. Additionally, a stronger fluorescent signal was also found for the V2 (NCs-PLGA-PEG) nanosystem in electroporated ovarian cancer cells. A very low fluorescent signal was detected when cells were not electroporated. Thus, we conclude that EP is favorable when a short time of incubation is required for the therapeutic protocol. Moreover, FA significantly enhanced toxicity against ovarian cancer cells. Our results are in good correspondence with other studies that also indicate functionalization as a promising factor in anticancer protocols [32,34].

### 3.4. Evaluation of PDT and EP-PDT

**Figure 6:**
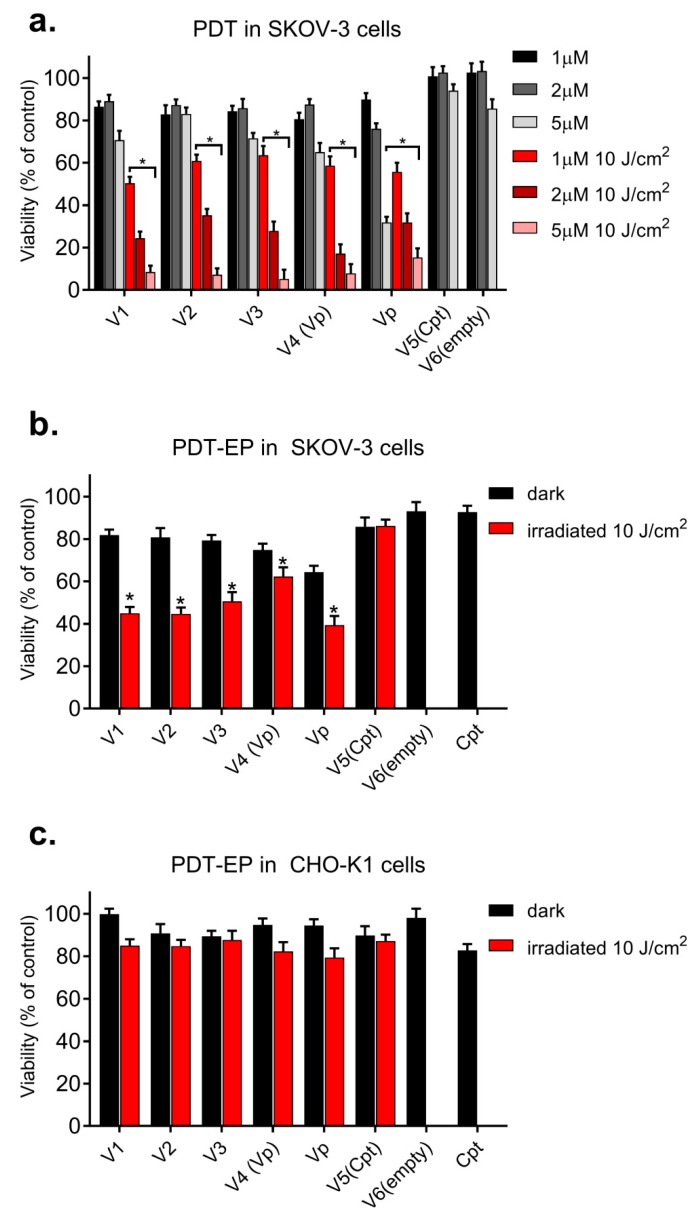
Photodynamic treatment (PDT) in SKOV-3 after 24 h of incubation with NCs (**a**) and photodynamic reaction facilitated by EP after 10 min exposition to nanosystems (*C*_VP_ = 1 μM) in SKOV-3 (**b**) and CHO-K1 (**c**) cells. The missing red bars for V6 and free Cpt represent non irradiated negative controls. See description of the NCs in Table 1. Data represented as means ± SD for minimum *n* = 3, where * *p* ≤ 0.05 was judged as statistically significant.

The results of photocytotoxicity studies for SKOV-3 cells are presented in Figure 6a. The cellular viability was assessed after 24 h of incubation with the NCs followed by irradiation. We found that the photodynamic effect increased proportionally to the concentration of the applied nanosystems. Longer time of incubation induced a significant decrease of cellular viability (60–80% decrease), in particular for the mixed cargo, proving the supportive anticancer effect of CisPt [35]. In Figure 6b,c, both types of cells were first electroporated and then exposed to NCs for 10 min.

After irradiation that followed EP, we observed a significant decrease of cellular viability in ovarian cancer cells in a short time, even in the lowest concentration of the applied NCs (*C*_VP_ = 1 µM).

This is in agreement with another study showing that EP enhances photodynamic reaction efficacy in the case of encapsulated drugs [12]. Thus, EP might be a promising and PDT-supporting tool for time-limited anticancer therapies. It is also worth noting that empty NCs (V6) with or without EP showed no toxic effect in SKOV-3 and CHO-K1 cultures, even at higher NC concentration (cell viability above 90%), proving the protective effect of the PLGA shell.

## 4. Conclusions

In this work, we demonstrate that a rationally designed double emulsion process leads to formulation of long-lasting, biocompatible, and “smart” NCs containing hybrid theranostic cargo with different hydrophobicity. We have designed, engineered, and characterized the effective polymeric nanocontainers (size ~200 nm) for efficient co-encapsulation of Cis-Pt and VP enabling drug delivery and synergistic anticancer activity via standard PDT and EP-enhanced PDT against human ovarian (SKOV-3) cancer cells. Loading hybrid cargo inside the oil core of NCs minimized its interaction with water environment, and thus, no drastic changes in physicochemical properties of the cytostatic drug and photosensitizer were observed. This feature is very favorable from the point of view of any bio-related application. Furthermore, EP with only 5 pulses and short time of loading exposition (10 min) enhanced delivery of encapsulated VP with all types of NCs. The highest photodynamic potential was noticed when VP was co-encapsulated with Cis-Pt and the “smart” NC functionalization with FA led to improved internalization by the SKOV-3 cells. Control CHO-K1 cells were significantly less sensitive to PDT, EP, and EP-PDT. In summary, the presented results reveal that the designed polymeric PLGA shells and colloidal cores in our NCs significantly improve PDT and EP-PDT and warrant future in vivo studies in animal models to fully prove their use for theranostic applications.

## Figures and Tables

**Table 1:** Physicochemical characteristics of VP and Cis-Pt co-loaded NCs with various PLGA shells.

System	Composition	D_H_ [nm]	PDI	ζ [mV]	*EE*_CisPt_	*EE*_VP_
V1	NCs-PLGA + VP + CisPt	193 ± 6	0.16 ± 0.01	−9 ± 1	92 ± 1	97 ± 3
V2	NCs-PLGA-PEG + VP + CisPt	187 ± 5	0.12 ± 0.01	−4 ± 1	88 ± 1	92 ± 1
V3	NCs-PLGA-FA + VP + CisPt	200 ± 7	0.20 ± 0.02	−15 ± 2	90 ± 2	95 ± 3
V4	NCs-PLGA-FA + VP	197 ± 7	0.22 ± 0.02	−16 ± 2	-	96 ± 3
V5	NCs-PLGA-FA + CisPt	194 ± 6	0.25 ± 0.02	−16 ± 2	92 ± 2	-
V6	NCs-PLGA-FA empty	189 ± 5	0.10 ± 0.01	−17 ± 3	-	-

## References

[1] Alexis F., Pridgen E., Molnar L.K., Farokhzad O.C. (2008). Factors affecting the clearance and biodistribution of polymeric nanoparticles. Mol. Pharm..

[2] Solans C., Izquierdo P., Nolla J., Azemar N., Garcia-Celma M.J. (2005). Nanoemulsions. Curr. Opin. Colloid Interface Sci..

[3] Bazylińska U., Kulbacka J., Wilk K.A. (2014). Dicephalic ionic surfactants in fabrication of biocompatiblenanoemulsions: Factors influencing droplet size and stability. Colloids Surf. A Physicochem. Eng. Asp..

[4] Fornaguera C., Feiner-Gracia N., Calderó N.G., García-Celma M.J., Solans C. (2016). PLGA nanoparticles from nano-emulsion templating as imaging agents: Versatile technology to obtain nanoparticles loaded with fluorescent dyes. Colloids Surf. B Biointerfaces.

[5] Fornaguera C., Dols-Perez A., Calderó N.G., García-Celma M.J., Camarasa J., Solans C. (2015). PLGA nanoparticles prepared by nano-emulsion templating using low-energy methods as efficient nanocarriers for drug delivery across the blood–brain barrier. J. Control. Release.

[6] Bazylińska U., Saczko J. (2016). Nanoemulsion-templated polylelectrolyte multifunctional nanocapsules for DNA entrapment and bioimaging. Colloids Surf. B Biointerfaces.

[7] Bazylińska U. (2017). Rationally designed double emulsion process for co-encapsulation of hybrid cargo in stealth nanocarriers. Colloids Surf. A Physicochem. Eng. Asp..

[8] McClements J.D. (2012). Advances in fabrication of emulsions with enhanced functionality using structural design principles. Curr. Opin. Colloid Interface Sci..

[9] Ahmed N., Michelin-Jamois M., Fessi H., Elaissari A. (2012). Modified double emulsion process as a new route to prepare submicron biodegradable magnetic/polycaprolactone particles for in vivo theranostics. Soft Matter.

[10] Salvadori C., Svara T., Rocchigiani G., Millanta F., Pavlin D., Cemazar M., Lampreht U., Sersa T.G., Tozon N., Poli A. (2017). Effects of electrochemotherapy with cisplatin and peritumoral IL-12 gene electrotransfer on canine mast cell tumors: A histopathologic and immunohistochemical study. Radiol. Oncol..

[11] Labanauskiene J., Gehl J., Didziapetriene J. (2007). Evaluation of cytotoxic effect of photodynamic therapy in combination with electroporation in vitro. Bioelectrochemistry.

[12] Kulbacka J., Kotulska M., Rembiałkowska N., Choromańska A., Kamińska I., Garbiec A., Rossowska J., Daczewska M., Jachimska B., Saczko J. (2013). Cellular stress induced by photodynamic reaction with CoTPPS and MnTMPyPCl5 in combination with electroporation in human colon adenocarcinoma cell lines (LoVo and LoVoDX). Cell Stress Chaperones.

[13] Zielichowska A., Saczko J., Garbiec A., Dubińska-Magiera M., Rossowska J., Surowiak P., Choromańska A., Daczewska M., Kulbacka J., Lage H. (2015). The photodynamic effect of far-red range phthalocyanines (AlPc and Pc green) supported by electropermeabilization in human gastric adenocarcinoma cells of sensitive and resistant type. Biomed. Pharmacother..

[14] Kulbacka J., Pucek A., Wilk K.A., Dubińska-Magiera M., Rossowska J., Kulbacki M., Kotulska M. (2016). The Effect of millisecond pulsed electric fields (msPEF) on intracellular drug transport with negatively charged large nanocarriers made of solid lipid nanoparticles (SLN): In vitro study. J. Membr. Biol..

[15] Kulbacka J., Pucek A., Kotulska M., Dubińska-Magiera M., Rossowska J., Rols M.P., Wilk K.A. (2016). Electroporation and lipid nanoparticles with cyanine IR-780 and flavonoids as efficient vectors to enhanced drug delivery in colon cancer. Bioelectrochemistry.

[16] Mutaliyeva B., Grigoriev D., Madybekova G., Sharipova A., Aidarova S., Saparbekova S., Miller R. (2017). Microencapsulation of insulin and its release using w/o/w double emulsion method. Colloids Surf. A Physicochem. Eng. Asp..

[17] Thompson K.L., Mable C.J., Lane J.A., Derry M.J., Fielding L.A., Armes S.P. (2015). Preparation of pickering double emulsions using block copolymer worms. Langmuir.

[18] Chen S., Yang K., Tuguntaev R.G., Mozhi A., Zhang J., Wang P.C., Liang X.J. (2016). Targeting tumor microenvironment with PEG-based amphiphilic nanoparticles to overcome chemoresistance. Nanomedicine.

[19] Kommineni N., Mahira S., Domb A.D., Khan W. (2019). Cabazitaxel-loaded nanocarriers for cancer therapy with reduced side effects. Pharmaceutics.

[20] Jang C., Lee J.H., Sahu A., Tae G. (2015). The synergistic effect of folate and RGD dual ligand of nanographene oxide on tumor targeting and photothermal therapy in vivo. Nanoscale.

[21] Bazylińska U., Wawrzyńczyk D. (2017). Encapsulation of TOPO stabilized NaYF_4_:Er^3+^,Yb^3+^ nanoparticles in biocompatible nanocarriers: Synthesis, optical properties and colloidal stability. Colloids Surf. A Physicochem. Eng. Asp..

[22] Bazylińska U., Zielińska K., Saczko J., Wilk K.A. (2012). Novel multilayer IR-786-loaded nanocarriers for intracellular delivering: Characterization, imaging, and internalization in human cancer cell lines. Chem. Lett..

[23] Gamper N., Stockand J.D., Shapiro M.S. (2005). The use of chinese hamster ovary (CHO) cells in the study of ion channels. J. Pharmacol. Toxicol. Methods.

[24] Méndez-Vilas A., Solano A. (2016). State of the art of smart polymers: From fundamentals to final applications. Polymer Science: Research Advances, Practical Applications and Educational Aspects.

[25] Azimi B., Nourpanah P., Rabiee M., Arbab S. (2014). Poly (lactic-co-glycolide) fiber: An Overview. J. Eng. Fibers Fabr..

[26] Callaghan R., Luk F., Bebawy M. (2014). Inhibition of the multidrug resistance P-glycoprotein: Time for a change of strategy?. Drug Metab. Dispos..

[27] Chen B., Le W., Wang Y., Li Z., Wang D., Ren L., Lin L., Cui S., Hu J.J., Hu Y. (2016). Targeting negative surface charges of cancer cells by multifunctional nanoprobes. Theranostics.

[28] Gutiérrez-Valenzuela C.A., Guerrero-Germán P., Tejeda-Mansir A., Esquivel R., Guzmán-Z R., Lucero-Acuña A. (2016). Folate functionalized PLGA nanoparticles loaded with plasmid pVAX1-NH36: Mathematical analysis of release. Appl. Sci..

[29] Lin P.C., Lin S., Wang P.C., Sridhar R. (2014). Techniques for physicochemical characterization of nanomaterials. Biotechnol. Adv..

[30] Zyuzin M.V., Honold T., Carregal-Romero S., Kantner K., Karg M. (2016). Influence of temperature on the colloidal stability of polymer-coated gold nanoparticles in cell culture media. Small.

[31] Kowalczuk A., Trzcinska R., Trzebicka B., Müller A.H.E., Dworak A. (2014). Loading of polymer nanocarriers: Factors, mechanisms and applications. Prog. Polym. Sci..

[32] Van der Steen S.C., Raavé R., Langerak S., Van Houdt L., Van Duijnhoven S.M., Van Lith S.A., Massuger L.F., Daamen W.F., Leenders W.P., Van Kuppevelt T.H. (2017). Targeting the extracellular matrix of ovarian cancer using functionalized, drug loaded lyophilisomes. Eur. J. Pharm. Biopharm..

[33] Siwowska K., Schmid R.M., Cohrs S., Schibli R., Müller C. (2017). Folate receptor-positive gynecological cancer cells: In vitro and in vivo characterization. Pharmaceuticals.

[34] Quarta A., Bernareggi D., Benigni F., Luison E., Nano G., Nitti S., Cesta M.C., Di Ciccio L., Canevari S., Pellegrino T. (2015). Targeting FR-expressing cells in ovarian cancer with Fab-functionalized nanoparticles: A full study to provide the proof of principle from in vitro to in vivo. Nanoscale.

[35] Lange C., Bednarski P.J. (2018). Evaluation for synergistic effects by combinations of photodynamic therapy (PDT) with temoporfin (mTHPC) and Pt(II) complexes carboplatin, cisplatin or oxaliplatin in a set of five human cancer cell lines. Int. J. Mol. Sci..

